# Role of radiotherapy in surgical approaches to pancreatic cancer treatment: A narrative review

**DOI:** 10.1002/ags3.70012

**Published:** 2025-03-07

**Authors:** Satoshi Yasuda, Minako Nagai, Kota Nakamura, Yasuko Matsuo, Masayuki Sho

**Affiliations:** ^1^ Department of Surgery Nara Medical University Nara Japan

**Keywords:** conversion surgery, neoadjuvant chemoradiotherapy, pancreatic adenocarcinoma, pancreatic surgery, radiation therapy

## Abstract

This review discusses the evolving role of radiotherapy (RT) in the surgical treatment of pancreatic ductal adenocarcinoma (PDAC). Despite advancements in multidisciplinary treatment, PDAC continues to present significant challenges in surgical treatment strategies. Neoadjuvant therapy, in combination with chemotherapy and RT, aims to improve patient outcomes by reducing tumor size, controlling local spread, and eradicating micrometastatic disease that cannot be detected at the time of diagnosis. Recent randomized trials have shown that both neoadjuvant chemoradiotherapy (NACRT) and neoadjuvant chemotherapy (NAC) improve surgical outcomes compared with upfront surgery. A network meta‐analysis integrating multiple trials demonstrated that NACRT significantly improves overall survival compared to NAC (HR: 0.79, 95% CI: 0.64–0.98). NACRT has also shown advantage in local tumor control. For locally advanced PDAC, the role of RT in conversion therapy is being actively investigated. The integration of RT in treatment regimens requires careful consideration of its therapeutic benefits against potential adverse effects. Although experimental studies suggest potential immunological benefits of RT, clinical validation remains incomplete. Recent advances in radiation delivery techniques have improved the therapeutic ratio, although further clinical validation is needed. The optimal sequence and combination of these treatment modalities with surgical strategies continue to be evaluated in ongoing clinical trials. This review synthesizes evidence from recent clinical trials and previous studies to evaluate the effectiveness, challenges, and potential of RT in PDAC treatment, aiming to inform both current clinical practice and future research directions.

## Graphical abstract

This review explores the role of radiotherapy (RT) in the multidisciplinary treatment of pancreatic ductal adenocarcinoma (PDAC), focusing on its integration with surgical strategies to improve resectability, local control, and survival outcomes while addressing potential challenges and future research directions.

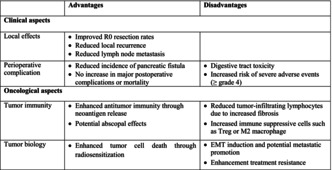

## 1 INTRODUCTION

Pancreatic ductal adenocarcinoma (PDAC) remains a highly aggressive disease with a dismal prognosis.^1^, ^2^ Managing this malignancy requires a multidisciplinary approach, incorporating advancements in diagnostic methods, perioperative treatment, radiotherapy (RT) techniques, and systemic therapy have led to steady improvements in patient outcomes.^1^, ^3^, ^4^ Initially, RT was employed for pain management and symptom palliation; however, technological innovations such as stereotactic body radiation therapy (SBRT) have enabled precise irradiation of tumors, contributing to local control and improved survival rates.^5^ Chemoradiotherapy (CRT) has further enhanced patient outcomes, especially in locally advanced or unresectable pancreatic cancers. Thus, RT plays an essential role in pancreatic cancer treatment.

The advent of neoadjuvant therapy (NAT) has shifted the treatment paradigm for pancreatic cancer by aiming to improve resectability by diminishing tumor volume, reducing local spread, and eradicating micrometastatic diseases present at the time of surgery to prolong overall survival (OS).^6^, ^7^, ^8^ Multidisciplinary treatments, including RT, for locally advanced pancreatic cancer (LAPC) have shown their potential to achieve conversion surgery (CS), facilitating radical resection.^9^ The combination of preoperative therapy and CS, particularly with CRT, requires careful consideration. The synergistic effects of chemotherapy and radiation therapy must be weighed against its potential adverse events. The selection of suitable patients, optimization of regimen, dose and sequence of irradiation, and management of adverse events are essential determinants of success.

This narrative review aims to clarify the current landscape of preoperative therapy in the surgical treatment of pancreatic cancer, with a particular focus on the role of RT. By reviewing the latest advances and assessing the potential advantages and disadvantages of CRT, we aim to contribute to the ongoing debate in pancreatic cancer treatment and provide insights to guide future research and clinical practice.

## 2 MATERIALS AND METHODS

### 2.1 Search strategy and data sources

A comprehensive systematic literature search was performed in PubMed (MEDLINE), Google Scholar, and Web of Science (accessed on February 1, 2024). The following search terms were used: (“Pancreatic Neoplasms “(Mesh) OR pancreas cancer* OR pancreatic neoplasm* OR pancreatic adenocarcinoma) AND (“Radiation Therapy” (Mesh) OR radiotherapy) AND (chemoradiation OR “Chemoradiotherapy” (Mesh) OR “Radiotherapy, Adjuvant” (Mesh) AND chemotherapy) AND (surgery OR “Surgical Procedures, Operative” (Mesh) OR conversion surgery OR pancreatectomy OR “pancreatic resection” OR pancreatoduodenectomy OR “distal pancreatectomy”). Additional relevant articles were identified through manual review of reference lists.

### 2.2 Inclusion and exclusion criteria

This review primarily focused on RCTs and prospective studies for evaluating key therapeutic strategies, while including high‐quality retrospective studies when they provided unique insights. Studies focused on CRT in pancreatic cancer comparing CRT and other treatments were included. Selection criteria included relevance, rigor of study design, and impact in journals. Exclusion criteria were: (i) irrelevant studies, (ii) editorials and letters, (iii) non‐English articles, (iv) case reports including fewer than 10 patients, (v) studies with insufficient data, and (vi) studies not focusing on CRT.

### 2.3 Data extraction

Two authors (S.Y. and M.S.) independently performed the literature assessment and data extraction on general study characteristics, CT regimens, OS after initial treatment and surgery, resection rate, and R0 resection rate. Disagreements were resolved through discussion.

## 3 RESULTS

### 3.1 Role of NACRT for resectable and borderline resectable PDAC

In the treatment of resectable and borderline resectable (BR) PDAC, NACRT synergistically enhances the cytotoxic effects of CT and RT, increasing the potential for substantial tumor shrinkage and achieving R0 resection, with reduced local recurrence risk. Moreover, systemic CT can target micrometastases often undetectable on imaging. Although postoperative adjuvant therapy with gemcitabine, S‐1, and modified FOLFIRINOX improves survival rates,^10^, ^11^, ^12^ one‐third of patients cannot complete treatment, highlighting the limitations of surgery followed by adjuvant CT.

#### 3.1.1 NACRT versus upfront surgery

A randomized controlled trial (RCT) conducted in South Korea in 2018^13^ demonstrated the efficacy of NACRT prior to surgery (Table 1). Gemcitabine with 54 Gy of radiation therapy showed a significantly improved R0 resection rate in the NACRT group in borderline resectable pancreatic cancer (BRPC) patients (82.4% vs. 33.3%, *p* = 0.010). The study also showed a significant reduction in lymph node metastasis (29.4% vs. 83.3%, *p* = 0.002) and median OS (21.0 months vs. 12.0 months, *p* = 0.028). The PREOPANC‐1 trial,^14^ a phase III RCT, provided robust evidence supporting NACRT. The trial demonstrated a significantly longer 5‐year survival rate for the NACRT group (OS median: 15.7 months vs. 14.3 months, *p* = 0.025) higher R0 resection rates (72% vs. 43%, *p* < 0.001), and lower lymph node metastasis (28% vs. 57%, *p* < 0.001) compared with the upfront surgery group. Similarly, the ESPAC5 trial^15^ demonstrated the advantages of NAT compared with upfront surgery. The NAT group exhibited a significant improvement in the 1‐year survival; however, the short follow‐up of 12 months limits the conclusions, and further follow‐up is necessary.

**TABLE 1:** RCTs of NACRT in PDAC.

Author year	Trial name	RCT phase	Pts (*n*)	Status	Treatment regimen (*n*)	Radiation (Gy/Fx)	Resection rate (%)	R0 rate (%)	Median OS (month)
NACRT vs. upfront surgery									
Golcher 2015		2	66	R	Gem + Cis + RT (33) US (33)	55.8	58 vs. 70	52 vs. 48	17.4 vs. 14.4
Casadai 2015		2	38	R	Gem ▸ Gem + RT (18) US (20)	54	61 vs. 75	25 vs. 38.9	22.4 vs. 19.5
Jang 2018		2, 3	50	BR	Gem + RT (18) US (20)	54/30	71 vs. 78	82.4 vs. 33.3*	21 vs. 12*
Versteijne 2022	PREOPANC‐1	3	246	R BR	Gem + RT (119) US (127)	36/15	61 vs. 72	72 vs. 43*	15.7 vs. 14.3*
Ghaneh 2023	ESPAC‐5	2	90	BR	NAT (57)^a^ US (33)	50.4/28	55 vs. 68	23 vs. 14	76% vs. 39%* (1‐yr OS)
NACRT vs. NAC									
Katz 2022	ALLIANCE A021501	2	126	BR	mFFX (x7) ▸ RT (56) mFFX (x8) (70)	SBRT 33–40/5 or HIGRT 25/5	51 vs. 58	74 vs. 88	17.1 vs. 29.8
Sugiura 2023	JASPAC 04	2	103	R	S‐1 + RT (51) GS (51)	50.4/28	90 vs. 92	98 vs. 89	37.7 vs. NR

Author year	Trial name (NCT number)	RCT phase	Pts (*n*)	Status	Treatment regimen (*n*)	Radiation (Gy/Fx)	Primary endpoint	Start year	Country
Ongoing clinical trials									
Gao 2019	BRPCNCC‐1 NCT03777462	3	243	BR	GnP GnP ▸ RT S‐1 + nab‐PTX ▸ RT	SBRT 37.5–40/5 SBRT 37.5–40/5	OS	2018	China
Janssen 2021A^b^	PREOPANC‐2	3	375	BR	Gem + RT FFX	36/15	OS	2018	Netherlands
	GABARNANC (JASPAC 07/EPOC A1601)	2, 3	50	BR	S‐1 + RT GnP	50.4/28	OS R0 Rate	2017	Japan
	PANDAS‐PRODIGE 44 NCT02676349	2	130	BR	FFX ▸ Cape + RT FFX	50.4 Gy	R0 Rate	2016	France
Bouchart 2023	STEREOPAC NCT05083247	2	256	BR	FFX/GnP ▸ FFX/GnP FFX/GnP ▸ RT ▸ FFX/ GnP	SBRT 35–55/5	R0 Rate DFS	2023	Belgium
	NCT05181605	2, 3	116	R	FFX ▸ RT US	NA	OS	2022	Spain
	NCT06259058	1, 2	96	BR	NALIRIFOX ▸ RT ▸ NALIRIFOX NALIRIFOX	SBRT 30/5	R0 Rate	2024	China

Abbreviations: BR, borderline resectable; Cape, capecitabine; Cis, cisplatin; DFS, disease‐free survival; FFX, FOLFIRINOX; Fx, fractions; Gem, gemcitabine; GnP, gemcitabine + nab‐paclitaxel; GS, gemcitabine + S‐1; HIGRT, hypofractionated image‐guided radiotherapy; mFFX, modified FOLFIRINOX; NA, not available; NAC, neoadjuvant chemotherapy; NACRT, neoadjuvant chemotherapy; NALIRIFOX, Nanoliposomal irinotecan‐based FOLFIRINOX; NAT, neoadjuvant therapy; NR, not reached; OS, overall survival; PDAC, pancreatic ductal adenocarcinoma; Pts, patients; PTX, paclitaxel; R, resectable; RCT, randomized controlled trial; RT, radiation therapy; SBRT, stereotactic body radiation therapy; US, upfront surgery; yr., year.

^a^ NAT (CT; FFX, GEM + CAPE, or CRT; CAPE + RT).

^b^ Interim report.

* *p* < 0.05.

#### 3.1.2 NACRT vs. NAC

Phase 2 RCTs comparing NACRT and NAC, including the JASPAC 04 trial,^16^ indicated both treatments were effective for RPC, with comparable 2‐year progression‐free survival (PFS) and 2‐year OS rates without significant differences in postoperative complications. However, the additive effects of RT requires further long‐term follow‐up.

The ALLIANCE A021501 trial^17^ evaluated NACRT and NAC with mFFX for BR pancreatic cancer, evaluating novel CT regimens. Patients received seven cycles of mFFX followed by RT, enrollment was terminated as the interim analysis criteria for the R0 resection rate were not met. Median survival times were 29.8 months for the NAC group and 17.1 months for the NACRT group. A subsequent analysis revealed that 59.0% of patients had inadequate radiation field coverage, highlighting technical challenges in radiation delivery.^18^

In real‐world settings, a multi‐institutional retrospective study compared NACRT and NAC, highlighting advantages in local control for NACRT, including higher R0 resection rates, and lower rates of lymph node metastasis and local recurrence.^19^, ^20^ However, NACRT was also associated with lower incidence of postoperative adjuvant CT, higher 90‐day mortality, and comparable median OS between the two groups.

While these individual trials failed to demonstrate the superiority of NACRT, a recent network meta‐analysis by He et al. of nine randomized trials (total 889 patients) demonstrated that NACRT significantly improved overall survival compared to NAC in resectable and borderline resectable pancreatic cancer (HR: 0.79, 95% CI: 0.64–0.98).^21^ However, this analysis also revealed increased treatment‐related adverse events and lower resection rates, suggesting the need for careful patient selection.

While individual trials and meta‐analyses have yielded conflicting results regarding the superiority of NACRT over NAC in the context of evolving CT and radiation therapy techniques, the need to determine an optimal combination of RT and CT through evaluation of RT is essential.

Several ongoing clinical trials, including PREOPANC‐2, BRPCNCC‐1, PANDAS‐PRODIGE 44, GABARNANCE, SUC2, and STEREOPAC will clarify the efficacy and safety of NACRT and NAC.

### 3.2 Role of CRT for unresectable locally advanced PDAC

The role of CRT in unresectable LAPC has been investigated in several studies (Table 2). The evolution of CRT has progressed from RT alone and BSC to more recent evaluations against modern CT regimens. The introduction of new CT regimens such as FFX and GnP has improved outcomes, making multidisciplinary treatments a crucial focus.

**TABLE 2:** RCTs and prospective studies CRT on conversion surgery for LAPC.

Author year	Trial name	RCT phase	Pts (*n*)	Treatment regimen (*n*)	Radiation (Gy/Fx)	Resection rate (%)	R0 rate (%)	Median OS (month)
CRT vs. BSC, radiation								
Moertel 1969			64	5‐FU + RT (32) RT (32)	35–40 40	NA	NA	10.4 vs. 6.3***
Moertel 1981			84	5‐FU + RT (28) 5‐FU + RT (31) RT (25)	40 60 60	NA	NA	10.6 vs. 10.1 vs. 5.7***
Shinchi 2002			31	5‐FU + RT (16) BSC (15)	50.4	NA	NA	13.2 vs. 6.4***
Cohen 2005		3	104	5‐FU + MMC + RT (55) RT (49)	59.4 59.4	NA	NA	8.4 vs. 7.1
CRT vs. CT								
Klaassen 1985			91	5‐FU + RT (47) 5‐FU (44)	40	NA	NA	8.2 vs. 8.3
GITSG 1988			43	5‐FU + RT (22) 5‐FU (21)	54	NA	NA	9.7 vs. 7.4***
Chauffert 2008		3	119	5‐FU + Cis + RT (59) Gem (60)	60	NA	NA	8.6 vs. 13.0***
Loehrer 2011			71	Gem + RT (34) Gem (37)	50.4	NA	NA	11.1 vs. 9.2***
Hammel 2016	LAP‐07	3	269	Gem ± erlotinib ▸ Cape + RT. (133) Gem ± erlotinib (136)	54/30	6 vs. 2.9	100 vs. 100	15.2 vs. 16.5
Prospective studies of CRT vs. CT on conversion surgery								
Hackert 2016		^a^	575^b^	FFX (125) Gem + RT (322) Others (128)	NA	61 vs. 46 vs. 52***	NA	16.0 vs. 16.5 vs. 14.5
Fietkau 2022^c^	CONKO‐007	3	167 169	Gem or FFX ▸ Gem + RT Gem or FFX	50.4/28	37 vs. 36	8 vs. 27^d^ ^,^ ***	15 vs. 15

Author year	Trial name (NCT number)	RCT phase	Pts (*n*)	Treatment regimen (*n*)	Radiation (Gy/Fx)	Primary endpoint	Start year	Country
Ongoing clinical trials								
	NCT01926197	3	172	mFFX ▸ RT mFFX	SBRT 40/5	PFS	2013	USA
Strauss 2019	SCALOP‐2 NCT02024009	2	289	Induction GnP ▸ GnP GnP ▸ RT + Cape GnP ▸ RT + Cape + nelfinavir	50.4/28 or 60/30	12‐month OS, PFS	2016	UK
Oar 2021	MASTERPLAN NCT04089150	2	120	FFX/GnP ▸ RT FFX/GnP	SBRT 40/5	12‐month local control	2019	Australia
	NCT04986930	2	92	FFX ▸ RT FFX	SBRT 35/5	1‐yr PFS	2021	Korea

Abbreviations: 5‐FU, 5‐fluorouracil; BSC, best supportive care; Cape, capecitabine; Cis, cisplatin; CRT, chemotherapy; FFX, FOLFIRINOX; Fx, fractions; Gem, gemcitabine; GnP, gemcitabine + nab‐paclitaxel; LAPC, locally advanced pancreatic cancer; mFFX, modified FOLFIRINOX; MMC, mitomycin; NA, not available; OS, overall survival; PDAC, pancreatic ductal adenocarcinoma; PFS, progression‐free survival; Pts, patients; RCT, randomized controlled trial; RT, radiation therapy; SBRT, stereotactic body radiation therapy; yr, year.

^a^ Prospective cohort study.

^b^ LAPC (435), URM (135).

^c^ Interim report.

^d^ R1 rate.

*** *p* < 0.05.

#### 3.2.1 Historical perspective of CRT for LAPC

Early studies by Moertel demonstrated that CRT, combined with 5‐fluorouracil (FU), significantly extended OS compared with RT alone (MSTs: 10.6 months vs. 5.7 months).^22^ Similarly, CRT with FU improved OS compared with BSC (13.2 months vs. 6.4 months).^23^ Trials comparing CRT with CT alone showed varying results. Klaassen et al. found no difference between FU‐based CRT and CT alone (MST: 8.3 months vs. 8.2 months), possibly due to low radiation dose (40 Gy).^24^ While a subsequent study demonstrated CRT's superiority (MST: 10.5 months vs. 8.0 months),^25^ Chauffert's research showed better survival with gemcitabine‐based CT compared to CRT using FU and cisplatin (MST: 13.0 months vs. 8.6 months).^26^ Loehrer's study later indicated survival benefits with gemcitabine‐based CRT over CT alone (MST: 11.1 months vs. 9.2 months).^27^ The pivotal LAP‐07 trial,^28^ comparing CRT with capecitabine and CT with gemcitabine and erlotinib, showed comparable OS (MST: 15.2 months vs. 16.5 months). Although historical studies demonstrated survival benefits of CRT compared to RT or BSC, comparisons of CRT and CT alone showed inconsistent results. Recent CT improvements necessitate further investigation of optimal combinations with RT for LAPC treatment.

#### 3.2.2 Conversion surgery for LAPC

CS for LAPC has gained attention as an emerging strategy decreasing tumors and allowing surgical resection through multidisciplinary treatment. It is clinically meaningful for the prognostic impact of converting inoperable into curatively resectable tumors. Multiple retrospective studies comparing CS reported substantial improvements in survival. A recent meta‐analysis including 1056 patients with LAPC,^9^ achieved an R0 resection rate of 84.4% and an MST of 32 months. Among these patients, 32% received FFX‐based CT, while 59.2% received gemcitabine‐based regimens, with 29.4% undergoing radiation therapy. The CS group (393 patients) versus the non‐resection group (1629 patients) showed favorable prognoses (hazard ratio: 0.55; 95% confidence interval: 0.45–0.66).

Hackert et al.^29^ prospectively analyzed 292 patients who underwent CRT or CT for locally advanced or metastatic unresectable pancreatic cancer and were eligible for conversion surgery. The resection rates were 46% for CRT with gemcitabine and 61% for CT with FFX, with MSTs of 16.5 and 16.0 months, respectively. These observations were obtained from the findings of observational analyses that compared patients deemed suitable for curative resection after effective preoperative treatment with those considered unsuitable for resection and were not derived from RCTs.

The optimal timing for conversion surgery in LAPC treatment remains undefined. The NCCN guidelines suggest considering conversion surgery after 4–6 months of induction chemotherapy in patients achieving disease stability, with additional radiation therapy recommended before surgical evaluation.^30^ However, beyond these guidelines, there is currently limited evidence regarding the optimal duration of treatment incorporating RT. Further studies are needed to determine the ideal timing and sequence of multimodal therapy including RT for conversion surgery in LAPC.

Preliminary results from the CONKO‐007 trial,^31^ investigating CRT on CS for LAPC, indicated that CRT with gemcitabine following induction CT increased pCR and R0 resection rates without significant OS benefits. These findings highlight the complexities of RT in conversion strategies and the need for further research to elucidate the optimal timing and role of CRT in conversion surgery. CRT for unresectable pancreatic cancer is performed to prolong patient survival and control local tumors. However, the benefits of radiation therapy remains to be fully clarified, particularly the direct contribution to long‐term survival. Continuous research is essential to determine the optimal application and efficacy of radiation therapy.

### 3.3 Advantages and disadvantages of additional RT to preoperative CT

The addition of RT to preoperative CT has multiple aspects, both in terms of clinical and oncologic advantages and disadvantages (Table 3). The decision to administer RT should be made after careful consideration of the balance between these factors as they affect both postoperative local control and long‐term prognosis.

**TABLE 3:** Advantages and disadvantages of RT in the treatment of PDAC.

	Advantages	Disadvantages
Clinical aspects		
Local effects	Improved R0 resection rates Reduced local recurrence Reduced lymph node metastasis	
Perioperative complication	Reduced incidence of pancreatic fistula No increase in major postoperative complications or mortality	Digestive tract toxicity Increased risk of severe adverse events (≥grade 4)
Oncological aspects		
Tumor immunity	Enhanced antitumor immunity through neoantigen release Potential abscopal effects	Reduced tumor‐infiltrating lymphocytes due to increased fibrosis Increased immune suppressive cells such as Treg or M2 macrophage
Tumor biology	Enhanced tumor cell death through radiosensitization	EMT induction and potential metastatic promotion Enhancement treatment resistance

Abbreviations: EMT, epithelial‐mesenchymal transition; PDAC, pancreatic ductal adenocarcinoma; RT, radiation therapy; Treg, regulatory T cell.

#### 3.3.1 Clinical advantages of RT

One of the most significant advantages of adding RT is enhanced local control. A recent network meta‐analysis has demonstrated that NACRT significantly improves overall survival compared to NAC (HR: 0.79, 95% CI: 0.64–0.98).^21^ The improvement is based on increased R0 resection rates, reduced local recurrence rates, and decreased lymph node metastases, all of which are crucial for long‐term survival.

RCTs have confirmed NACRT improved R0 resection rates for RPC or BRPC, as demonstrated by Jang et al.^13^ (82.4% vs. 33.3%, *p* = 0.01) and the PREOPANC‐1 trial^14^, ^32^ (72% vs. 43%, *p* < 0.001). Nagakawa et al. demonstrated NACRT significantly reduced lymph node metastasis (37.8% vs. 66.0%, *p* < 0.001) and local recurrence rates (20.4% vs. 44.6%, *p* = 0.002) compared to NAC.^19^ These findings were supported by a large‐scale NCDB analysis comparing 3185 NACRT patients with 3751 NAC patients, which showed improved R0 rates (86.1% vs. 80.0%, *p* < 0.001) and lymph node metastasis rates (38.9% vs. 60.1%, *p* < 0.001) with NACRT.^20^ These results consistently demonstrate the local control benefits of NACRT, though this analysis demonstrated a higher 90‐day mortality in the NACRT group (4.8% vs. 2.7%, *p* < 0.001), and RT‐induced arteritis has been suggested as a potential risk factor for postoperative hemorrhage. However, it should be noted that specific complication data were not provided in this analysis, and major randomized trials including PREOPANC‐1 and JASPAC 04 have shown that NACRT was not associated with increased postoperative mortality.^16^, ^33^ Recent RCTs comparing NACRT and NAC have shown varying results regarding local control. The ALLIANCE trial and JASPAC 04 trial demonstrated comparable R0 resection rates between treatment groups.^16^, ^17^ However, in LAPC patients, the CONKO‐007 trial showed significantly lower R1 rates with CRT (3.0% vs. 9.6%, *p* = 0.0085).^31^ Additionally, Krishnan et al.^34^ reported improved local recurrence‐free survival rate with high‐dose radiation therapy (>70 Gy) compared to standard dose of 54 Gy.

Among the complications of pancreatic surgery, pancreatic fistulas are critical. As we previously reported,^35^ preoperative treatments, including NACRT decrease pancreatic fistulas, attributed to enhancement of fibrosis and hardening in the pancreatic parenchyma. The PREOPANC‐1 trial confirmed preoperative treatment effectively reduces pancreatic fistulas.^33^ Large‐scale analysis comparing NACRT and NAC showed that NACRT significantly reduced pancreatic fistulas (3.5%) compared to both upfront surgery group (20.1%) and NAC (11.2%).^27^ Recent large‐scale studies have addressed surgical concerns regarding RT's impact on operative difficulty and complications. The PREOPANC‐1 trial demonstrated that preoperative CRT did not increase major complications (37.9% vs. 30.6%, *p* = 0.400), postpancreatectomy hemorrhage (9.1% vs. 5.1%, *p* = 0.352), or intra‐abdominal infections (12.1% vs. 10.2%, *p* = 0.800).^33^ Intraoperative blood loss remained comparable between the groups (median: 900 mL vs. 900 mL, *p* = 0.695), and postoperative mortality showed no significant difference (3.0% vs. 4.1%, *p* = 1.000). These findings suggest that while RT induces local tissue changes, experienced surgical teams can safely manage these alterations without increased technical difficulty or perioperative risk. Initial concerns about increased overall complications and postoperative mortality have been mitigated by RCTs^33^ and meta‐analyses^36^ reporting no significant impacts.

#### 3.3.2 Clinical disadvantages of RT

The addition of RT to chemotherapy presents several clinical challenges that warrant careful consideration. Recent clinical trials revealed the safety profile of NACRT. The PREOPANC‐1 trial^14^ and ALLIANCE trial^17^ demonstrated comparable rates of grade 3 or higher adverse events between NACRT and NAC groups. Common adverse events include gastrointestinal toxicity, hematological abnormalities, and general fatigue. However, Loehrer et al.^37^ reported a higher incidence of grade 4 adverse events in the NACRT group, emphasizing careful toxicity management.

SBRT is a promising treatment option with lower complication rates and higher local control than conventional RT.^5^ However, a recent study highlighted technical challenges in radiation quality assurance, reporting inadequate radiation field coverage in 59.0% of patients.^18^

The impact of outpatient radiation therapy varies depending on patients' social circumstances. Younger employed patients may benefit from continuing treatment on an outpatient basis, while elderly patients or those living far from treatment centers may face physical and economic burdens from frequent hospital visits. Treatment planning should consider individual patient circumstances and preferences.

#### 3.3.3 Oncological advantages

**FIGURE 1:**
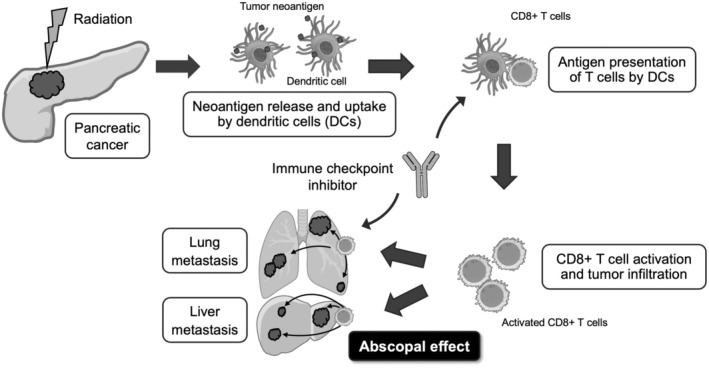
Radiation therapy (RT)‐induced immune responses and abscopal effect. RT triggers the release of tumor neoantigens, leading to enhanced uptake by dendritic cells and subsequent activation of CD8+ T cells. This immune response can affect distant metastases through the abscopal effect. Furthermore, immune checkpoint inhibitors may augment such efficacy.

Pancreatic cancer has long been recognized for its low immunogenicity,^38^ posing challenges for immunotherapy interventions. Tumors with such attributes may require immune activation across multiple stages of the cancer immunity cycle.^39^ RT has the potential to activate tumor immunity by releasing tumor neoantigens that can trigger an immune response and induce a tumor‐specific cytotoxic T lymphocyte reaction. The immune activation by RT may lead to the regression of metastases that are not directly irradiated (known as the abscopal effect), which could be beneficial for controlling occult PDAC micrometastases (Figure 1).

Recent studies have provided new insights into the mechanisms of RT‐induced immune responses. Yamamoto et al. demonstrated that high‐dose local irradiation significantly enhances the abscopal effect in a mouse pancreatic cancer model treated with an anti‐CTLA‐4 antibody.^40^ These results suggest that radiation can alter the immune microenvironment and improve the systemic response of tumors beyond the direct irradiation site. This findings is consistent with previous research indicating that combining immune checkpoint inhibitors with radiation can enhance the abscopal effects in various cancer types.^41^, ^42^ Additionally, NACRT has been shown to reduce M2 macrophage infiltration in the tumor microenvironment of female patients, leading to enhanced anti‐tumor immunity.^43^ While this observation was limited to females, it provides valuable clinical evidence of the immunomodulatory effects of radiotherapy in pancreatic cancer and warrants further investigation in larger cohorts. Radiation promotes the release of danger signals such as high mobility group box 1 (HMGB‐1) from PDAC cells, facilitates phagocytosis of tumor cells by dendritic cells, and enhances tumor antigens to CD8 T cells. These findings imply that high doses of radiation contribute to changes in the microenvironment, although the precise mechanism remains unclear. The combination of radiation and immunotherapy has been explored in several clinical trials. A phase I/II trial (NCT02305186) evaluates neoadjuvant chemoradiation with pembrolizumab in resectable or borderline resectable disease. Two phase II trials investigate immune‐based combination therapies in pancreatic cancer: one (NCT03161379) investigates cyclophosphamide, nivolumab, and GVAX pancreas vaccine combined with SBRT in borderline resectable disease, while another trial (NCT02648282) evaluates a similar regimen replacing nivolumab with pembrolizumab in locally advanced pancreatic cancer. Furthermore, the expression of CA9, which increases in a hypoxic environment, is closely associated with radiosensitivity, and CA9‐positive cases in the NACRT group showed significantly better prognosis compared to those in the upfront surgery group.^44^ The identification of such molecular markers may contribute to optimal patient selection for CRT.

#### 3.3.4 Oncological disadvantages

RT can also negatively affect tumor immunity. High‐dose irradiation promotes tumor fibrosis and inhibit T‐cell infiltration, potentially diminishing the ability of the immune system to combat cancer.^45^ Furthermore, RT increases immunosuppressive cells such as regulatory T cells (Tregs) and M2 macrophages, aiding tumor evasion of the immune response.^45^, ^46^ Although increased CD8+ T‐cell infiltration following high‐dose radiation has been associated with favorable outcomes in various malignancies,^47^ the dense stromal environment of PDAC complicates the immune response.

Additionally, RT may promote metastasis by inducing epithelial‐to‐mesenchymal transition (EMT) through cancer‐associated fibroblasts. Mueller et al. showed that post‐SBRT patients exhibit increased expression of EMT‐related genes and that radiation‐induced metastasis is associated with ADAM10‐mediated fibrosis promotion.^46^

Treatment resistance is also a critical concern. Studies have shown that RT induces autophagy,^48^ leading to radiation resistance. RT‐enhanced invasive capacity of pancreatic cancer cells is associated with matrix metalloproteinase‐2,^49^ whose inhibition suppresses the invasiveness.

While RT demonstrates promising immunological benefits, careful consideration must be given to its potential adverse effects such as EMT induction and treatment resistance. These findings underscore the importance of patient selection and timing of RT in pancreatic cancer treatment.

The limitations of this review include publication bias and variability in study design, target population, and treatment protocols, which affect the generalizability of these findings. Since pancreatic cancer treatments are rapidly evolving, ongoing clinical trials may provide additional insights.

## 4 CONCLUSIONS

The utilization of CRT in preoperative treatment is important in the multidisciplinary treatment of PDAC. CRT has demonstrated potential in improving resectability and OS by reducing tumor volume, improving local control, and addressing systemic micrometastases. However, CRT requires careful patient selection to strike a balance between benefits and adverse effects. Major concerns include toxicity, potential promotion of tumor metastasis, and immune suppression, despite its advantages in tumor control and immunogenic response.

Future research should focus on several critical areas. First, establishment of predictive biomarkers for RT response, including molecular markers and imaging biomarkers, will enable more precise patient selection. Second, the optimal timing of conversion surgery for LAPC following CRT needs to be clarified through prospective trials, particularly focusing on the duration of preoperative therapy and criteria for surgical intervention. Third, investigation of combination strategies between RT and immunotherapy, including checkpoint inhibitors, will be crucial for enhancing treatment efficacy while managing potential adverse effects.

In clinical practice, this review has revealed two critical perspectives regarding the application of CRT. First, while consistent evidence demonstrates improved local control with CRT, its impact on long‐term survival requires further validation. Although a recent network meta‐analysis suggested survival benefits, the lack of definitive superiority in individual clinical trials necessitates careful interpretation of these findings. Second, the safety profile of CRT has been well‐characterized: while it reduces postoperative pancreatic fistula rates without increasing overall surgical complications, the potential for severe adverse events during preoperative treatment demands careful patient selection and vigilant monitoring. These findings emphasize the importance of optimizing treatment protocols, including CRT timing, TNT implementation, RT modalities, CT regimens, and dosage, to maximize therapeutic efficacy. Ongoing clinical trials are critical in elucidating the role of CRT and devising treatment strategies for PDAC, contributing to a more personalized, evidence‐based approach.

## AUTHOR CONTRIBUTIONS

**Satoshi Yasuda:** Conceptualization; data curation; formal analysis; investigation; project administration; writing – original draft; writing – review and editing. **Minako Nagai:** Methodology. **Kota Nakamura:** Investigation. **Yasuko Matsuo:** Writing – review and editing. **Masayuki Sho:** Conceptualization; supervision; writing – review and editing.

## FUNDING INFORMATION

This study received no external funding.

## CONFLICT OF INTEREST STATEMENT

Masayuki Sho is an Associate Editor of *Annals of Gastroenterological Surgery*. The other authors declare no conflict of interest.

## ETHICS STATEMENT

Approval of the research protocol by an Institutional Reviewer Board: N/A

Informed Consent: N/A

Registry and the Registration No. of the study/trial: N/A

Animal Studies: N/A

## Data Availability

No new data was created or analyzed in this study, and data sharing does not apply to this paper. All relevant data to this study are contained within the article.
